# COMP and TSP-4 interact specifically with the novel GXKGHR motif only found in fibrillar collagens

**DOI:** 10.1038/s41598-018-35447-8

**Published:** 2018-11-21

**Authors:** Jan M. Gebauer, Anna Köhler, Helen Dietmar, Monika Gompert, Ines Neundorf, Frank Zaucke, Manuel Koch, Ulrich Baumann

**Affiliations:** 1 Institute of Biochemistry, University of Cologne, Cologne, Germany; 2 Center for Biochemistry, Faculty of Medicine and University Hospital Cologne, University of Cologne, Cologne, Germany; 3 Dr. Rolf Schwiete Research Unit for Osteoarthritis, Orthopedic University Hospital Friedrichsheim gGmbH, Frankfurt, Germany; 4 Institute for Dental Research and Oral Musculoskeletal Biology, Faculty of Medicine and University Hospital Cologne, University of Cologne, Cologne, Germany

## Abstract

COMP (cartilage oligomeric matrix protein) is a member of the thrombospondin family and forms homopentamers as well as mixed heterooligomers with its closely related family member TSP-4. COMP is long known to bind to collagens and to influence collagen fibril formation. Recent work indicates that already intracellular interaction with collagen is important for collagen secretion. However, the exact binding site of COMP on the collagen triple helix has not been described up to now. In this study we have identified a GXKGHR motif on the collagen II helix to bind to COMP, using a recombinantly expressed collagen II peptide library. This binding sequence is conserved throughout evolution and we demonstrate that TSP-4 binds to the same sequence. The identified binding motif overlaps with the recognition sites of many other collagen-binding partners (e.g. PEDF, Heparin) and also spans the lysine residues, which form collagen cross-links. COMP might thereby protect collagen helices from premature modification and cross-linking. Interestingly, this motif is only found in classical fibrillar collagens, although COMP is known to also bind other types. This might indicate that COMP has a unique interface for fibrillar collagens, thus making it an interesting target for the development of antifibrotic drugs.

## Introduction

Collagens are an essential component of the extracellular matrix (ECM) of all higher organisms. 28 family members are currently recognized, numbered with roman numbers from I to XXVIII^1^. Collagens always form trimers and the individual polypeptides are called α chains. Some collagens form homotrimers consisting of three identical polypeptides, while others form heterotrimers with two or three different α chains. All of them share as defining feature the so-called collagen triple helix; a structural element that is formed by three individual polypeptide chains (reviewed in^1–3^). The three-dimensional structure of this extended, non-globular domain requires a glycine in every third position, leading to a repetition of the triplet motif Gly-Xaa-Yaa. Additionally, the Xaa and Yaa position are often prolines and hydroxyprolines, respectively (approx. 27% and 38%). Biosynthesis of collagen is a complicated process involving extensive post-translational modifications^4^. After secretion into the endoplasmic reticulum (ER), a large network of modifying enzymes like proline-hydroxylase or glycotransferases as well as molecular chaperones, such as protein disulphide isomerase, binding immunoglobulin protein (BIP), or heat shock protein 47 (HSP47)^4,5^ are involved in the formation of a properly modified and folded trimeric procollagen molecule that is secreted via the Golgi compartment to the extracellular space.

Collagens form a great diversity of superstructures. Besides the well-known collagen fibrils, they also form hexagonal networks (VIII & X), beaded filaments (VI), basement membrane networks (IV), and anchoring fibrils (VII). Some collagens are even membrane bound (XIII, XVII, XXIII, XXV). Collagens have a vast variety of functions, as demonstrated by their causative role in various inherited diseases, with *Epidermolysis bullosa* (e.g. collagen VII) and *Osteogenesis imperfecta* (e.g. collagen I) being amongst the most well-known collagen linked diseases. The interactions of the collagen helix with other extracellular proteins is very important for the understanding of the extracellular matrix and its related diseases; however, to date the exact nature of many interactions is only poorly understood.

This is partly because collagen domains are difficult to express recombinantly and even more difficult to express as fragments. For other protein interactions, truncations and site directed mutagenesis normally lead to a general idea of a binding interface. Ideally, this knowledge is further used to crystallize the minimal binding complex, leading to detailed understanding of the interaction. While this method is very suitable for the non-collagenous side of the interaction, collagenous domains are difficult to manipulate. Breaking collagenous domains into pieces is not trivial, as the triple helical structure needs to be retained. Current techniques involve the chemical synthesis of collagen model peptides with a host-guest-approach. Typically, the sequence of interest is flanked by approximately six glycine-proline-hydroxyproline (GPO) repeats on both sides. The GPO repeats serve as a host and facilitate the formation of a triple helical collagen structure. However, only peptides of a certain length can be efficiently produced and the synthesis is time consuming and expensive. With collagenous domains of more than 300 triplets, screening the whole domain with overlapping fragments is only feasible for the most abundant collagens. Indeed peptide libraries, called “collagen toolkits”, are only available for collagen II and III^6^.

Besides chemical synthesis, collagen model peptides can also expressed recombinantly. Often trimerisation domains, like the T4 foldon domain, are used in these systems in order to stabilise the truncated triple helix^7^. The collagenous part is normally designed in the same host-guest approach mentioned above. Typically expressed in *E. coli*, these fragments can be much longer and are produced at lower costs. However, incorporation of hydroxyproline in *E. coli* is difficult, and the use of eukaryotic cell lines to overcome this problem increases the cost and time significantly. Recent improvements in the post-translational generation of hydroxyproline in prokaryotes^8^ might alleviate this problem. However, to our knowledge, recombinantly expressed collagen peptide libraries were not systematically used before this study.

**Figure 1:**
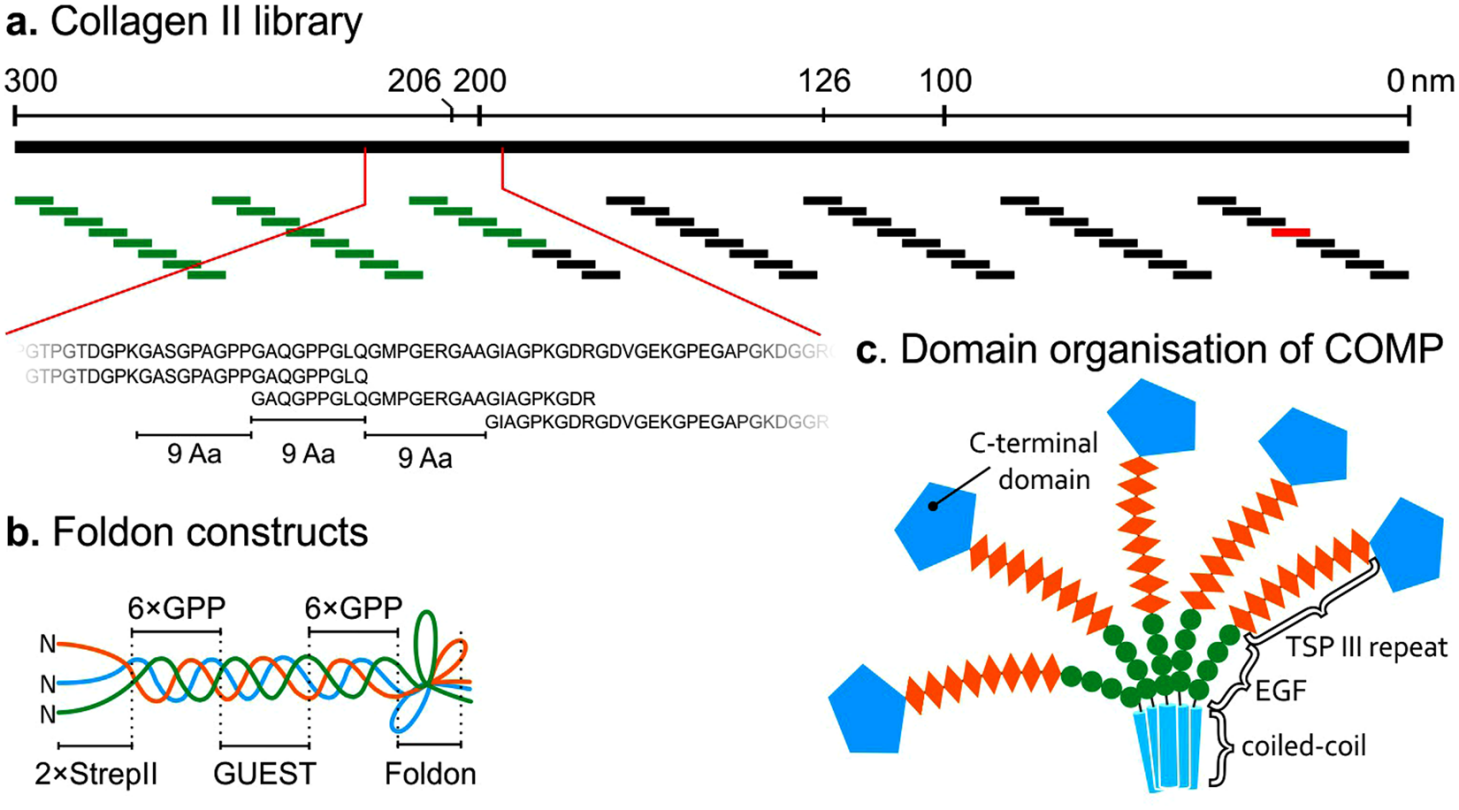
Organisation of the collagen library and protein constructs. (**a**) The 300 nm long collagen II triple helical domain was divided into 56 overlapping peptide sequences. The amino acid (Aa) sequences were backtranslated and optimised for *E. coli* expression^43^. The current library covers the N-terminal region of collagen II covering the sequence stretch between _210_GPMGPM to GEDGRP_587_ of the collagen helix (peptides in green/Table 1). The red marked peptide (pep52) was also expressed later in the study. (**b**) The sequences were inserted into the GUEST position of a vector coding for a GPP collagen host sequence with a C-terminal foldon domain. (**c**) COMP is a pentameric protein with a multi-domain structure.

One of the important interaction partners of collagens in cartilage is the cartilage oligomeric matrix protein (COMP) a member of the thrombospondin family of proteins, also referred to as TSP-5. COMP forms homopentamers induced by an N-terminal coiled-coil domain, which is followed by four epidermal growth factor-like domains (EGF), eight thrombospondin type III repeats, and a C-terminal COMP (TC) domain (Fig. 1c)^9^. In healthy situations COMP has been described to fulfil numerous functions by cross-linking various components in the extracellular matrix and might therefore play an important role in the organisation of the different matrix protein networks^9^. In addition to other extracellular matrix proteins (for review see^9^), COMP is described to bind directly to collagen type I and II^10^, collagen IX^11^, as well as collagen XII and XIV^12^. Binding of pentameric COMP to the fibrillar collagens leads to an increased rate of fibril formation *in vitro*^13^, while the interaction of COMP with collagens XII and XIV may be important for the dermal-epidermal junction^12^. Recently, the importance of intracellular COMP/collagen interaction for collagen secretion was described^14^, a function also attributed to HSP47^15^.

Mutations in COMP can lead to skeletal disorders in humans, ranging from mild to severe forms of multiple epiphyseal dysplasia (MED) or pseudoachondroplasia (PSACH)^16^. Most disease-causing mutations are not secreted and the pathology is believed to be caused by a co-retention of fibrillar collagen. For some mutations, however, the secretion is normal and for one particular mutation (pH587R) an aberrant extracellular fibril formation is observed^17^. Interestingly, this mutation (and others), lie in the C-terminal domain of COMP^18^, which is responsible for the collagen binding.

Despite this well documented functional importance of the collagen-COMP interaction, the exact binding interface on the collagen triple helix is unknown up to now. In this study, we identified for the first time the direct binding epitope for COMP and TSP-4 on the collagen helix.

## Results

### Generation of the N-terminal collagen library

From electron micrographs it is known that COMP binds to four distinct sites, located at approximately 0, 126, 206, and 300 nm distance from the C-terminus of the triple helix^10^. To elucidate the N-terminal binding site of COMP, we generated a foldon stabilised, *E. coli* expressed, collagen model peptide library spanning the first 100 nm of collagen II (Fig. 1 and Table 1). Although the design of the library was inspired by the collagen II toolkit^6^, by using a foldon tag and a prokaryotic expression system, we hoped to reduce costs and increase speed for identification and characterisation of binding sites. Furthermore, the existence of a foldon tag and an affinity tag enables certain biochemical assays (e.g. biolayer interferometry), difficult to perform with synthesised peptides. The library was generated using the golden gate assembly method^19,20^, which uses Type IIS restriction endonuclease. These enzymes cut outside of their recognition sequence. With a proper design of the vectors and PCR primers (respectively inserts) the recognition sites can be excluded from the final product. Cycling between cutting and ligation steps will thus accumulate the only stable product. In our study we modified an existing pET vector^21^ already containing several GPP repeats, an N-terminal 2xStrep tag and a C-terminal foldon domain. Via a central XhoI site, we introduced the necessary acceptor sites (pCMP-3b, Fig. S2). This vector together with synthetically synthesised DNA oligos allowed rapid cloning, with nearly 100% positive clones. The purified peptides run as a partially SDS-stable trimer on SDS-PAGE.

**Table 1:** Sequences of the collagen II N-terminal foldon library.

No	Sequence
1	GPMGPMGPRGPPGPAGAPGPQGFQGNP
2	GPQGFQGNPGEPGEPGVSGPMGPRGPP
3	GPMGPRGPPGPPGKPGDDGEAGKPGKA
4	GEAGKPGKAGERGPPGPQGARGFPGTP
5	GARGFPGTPGLPGVKGHRGYPGLDGAK
6	GYPGLDGAKGEAGAPGVKGESGSPGEN
7	GESGSPGENGSPGPMGPRGLPGERGRT
8	GLPGERGRTGPAGAAGARGNDGQPGPA
9	GNDGQPGPAGPPGPVGPAGGPGFPGAP
10	GGPGFPGAPGAKGEAGPTGARGPEGAQ
11	GARGPEGAQGPRGEPGTPGSPGPAGAS
12	GSPGPAGASGNPGTDGIPGAKGSAGAP
13	GAKGSAGAPGIAGAPGFPGPRGPPGPQ
14	GPRGPPGPQGATGPLGPKGQTGEPGIA
15	GQTGEPGIAGFKGEQGPKGEPGPAGPQ
16	GEPGPAGPQGAPGPAGEEGKRGARGEP
17	GKRGARGEPGGVGPIGPPGERGAPGNR
18	GERGAPGNRGFPGQDGLAGPKGAPGER
19	GPKGAPGERGPSGLAGPKGANGDPGRP
20	GANGDPGRPGEPGLPGARGLTGRPGDA
21	GLTGRPGDAGPQGKVGPSGAPGEDGRP
52	GEAGEPGERGLKGHRGFTGLQGLPGPP

All peptide sequences are presented in an NStrep-(GPP)_6_-GUEST-(GPP)_6_-foldon context. Underlined sequences are unique and are only present in one peptide.

### COMP binds to discrete peptides on the collagen library

**Figure 2:**
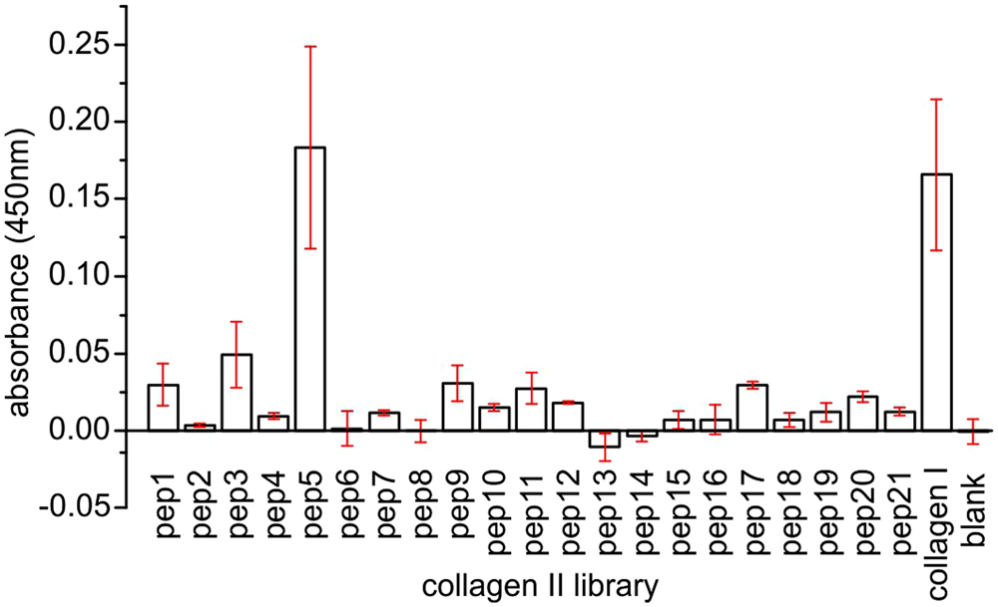
Binding of COMP to the N-terminal collagen II foldon library. Foldon stabilised collagen mimetic peptides and rat-tail collagen I as positive control were coated to an ELISA plate and incubated with 200 nM pentameric COMP. Binding was detected with a specific anti-COMP antibody. Bars indicate standard deviation of triplicates. The peptide sequences are depicted in Table 1.

**Figure 3:**
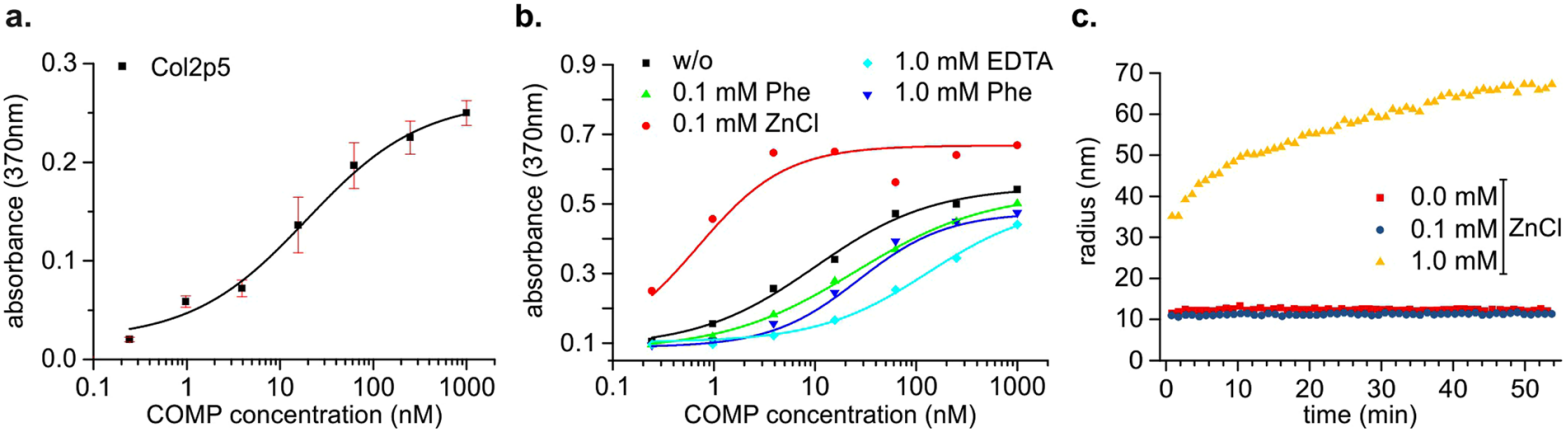
Binding of COMP to peptide 5 and the dependency of Zn^2+^. Peptide 5 of the collagen II foldon library was coated to an ELISA plate and incubated with COMP protein (**a**) or with COMP in interaction buffer containing indicated chemical compounds (**b**). Binding was detected with a specific anti-COMP antibody. Bars indicate standard deviation of triplicates. The peptide sequence is depicted in Table 1. The calculated K_D_ for the pep5 interaction in (**a**) is 19.3 ± 12.4 nM. (**c**) The radius of particles (obtained by cumulant analysis) measured of a COMP solution with addition of the indicated ZnCl ions were monitored over time using dynamic light scattering. *Phe:* 1,10-phenanthroline.

To elucidate the binding site of COMP on the collagen II helix we incubated pentameric COMP at 200 nM concentration with our generated collagen II foldon library (Fig. 2). Only one peptide (pep5) showed a similar signal for the COMP binding in comparison with the collagen I interaction, which served as a positive control. To confirm our finding and get proper binding affinities we coated the peptide 5 and incubated it with indicated concentration of COMP (Fig. 3a). The dissociation constant for COMP and pep5 (19.3 ± 12 nM) was slightly higher than reported for collagen II (1.72 ± 0.16 nM)^10^. It is worthwhile mentioning, that COMP (as a pentamer) will exhibit significant avidity effect in solid-phase binding assays, which are dependent of the density of the coated binding partner.

COMP is described to interact with collagen I and II in a zinc dependent fashion. In our experiments, adding zinc was not strictly necessary for the interaction, but increased the affinity towards the peptides (Fig. 3b). However, we also noted an increase in background binding with 0.1 mM zinc. If zinc is important for the interaction, recombinantly expressed COMP needs to already carry zinc as we observe binding even in pure TBS. Following this line of argument, additional zinc would only fill empty binding pockets in COMP, thus increasing the active population of COMP in the solution. To test this hypothesis, we incubated COMP with 1,10-phenanthroline a high-affinity zinc chelator and EDTA a more general chelator of divalent cations. Interestingly, phenanthroline does not reduce the binding compared to pure TBS, indicating that there is no “pre-charged zinc” present or at least not necessary for the interaction. EDTA, however, reduced the binding significantly (Fig. 3b), but this can easily be explained by the necessity of calcium ions for the overall structural integrity of COMP’s TSP repeats. If we incubate COMP with various concentrations of zinc and observe the hydrodynamic radius via DLS, we could not detect any difference between 0.0 mM and 0.1 mM ZnCl, but COMP starts to aggregate dramatically upon addition of 1 mM ZnCl (Fig. 3c).

### The minimal binding site of COMP on the N-terminus on collagen I is GPKGHR

**Figure 4:**
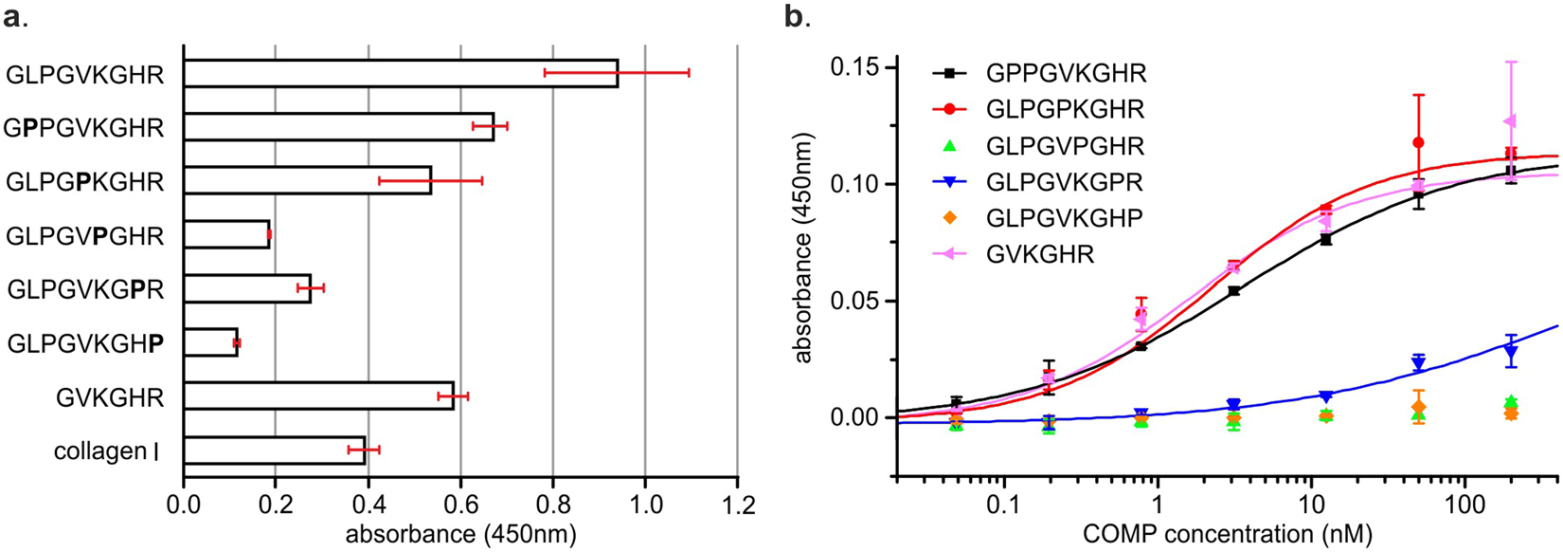
Characterisation of important residues for COMP binding. Amino acids, with the exception of proline or glycine, were exchanged to proline (bold indicated). COMP was incubated either at 400 nM (**a**) or at indicated concentrations (**b**). Binding was detected with a specific anti-COMP antibody. Bars indicate standard deviation of triplicates.

The peptide 5 of the library has a total length of 27 amino acids. However, as the adjacent peptides 4 and 6 are not interacting, the amino acids responsible for COMP interactions has to be in or at least part of the unique region of pep5. We first tried, whether the unique region of pep5 “GLPGVKGHR” is the binding epitope for COMP and indeed this motif was sufficient for binding, as shown by one-point measurements (Fig. 4a). To further narrow down the exact prerequisites for COMP binding, we systematically exchanged all none-glycine amino acids in the GLPGVKGHR motif to proline residues (Table 2). Interestingly, neither the exchange of the leucine nor the valine did reduce the affinity significantly (Fig. 4b). The exchange of the arginine or the lysine residue, however, reduced the binding affinities below measurable values. The exchange of the histidine showed an intermediate effect.

**Table 2:** Peptides used for further analysis of the minimal binding site.

Sequence	Apparent K_D_ (nM)	Melting point (°C)
GARGFPGTPGLPGVKGHRGYPGLDGA	19.3 ± 12.4	36.1 ± 0.5
GLPGVKGHR	n.d.	48.2 ± 0.6
G**P**PGVKGHR	3.57 ± 0.37	54.2 ± 0.7
GLPG**P**KGHR	2.26 ± 0.39	52.5 ± 0.6
GLPGV**P**GHR	n.a.	51.1 ± 0.3
GLPGVKG**P**R	496 ± 3203	n.d.
GLPGVKGH**P**	n.a.	47.9 ± 0.4
GVKGHR	1.61 ± 0.29	56.5 ± 0.6

The sequence unique to peptide 5 is underlined and mutations in comparison to the wt sequence are marked in bold. All peptide sequences are presented in an N-Strep-(GPP)_6_-GUEST-(GPP)_6_-foldon context. Melting points were determined by CD spectroscopy at 210 nm. Exemplary spectra can be found in the Fig. S1.

(n.d. - not determined; n.a. - not applicable/no measurable signals observed to fit K_D_ values).

Knowing that the leucine is not important for binding, we tried to further narrow down the interaction motif to GVKGHR and observed a similar binding as to original peptides (Fig. 4).

### Collagen II has a second binding site close to the C-terminus

**Figure 5:**
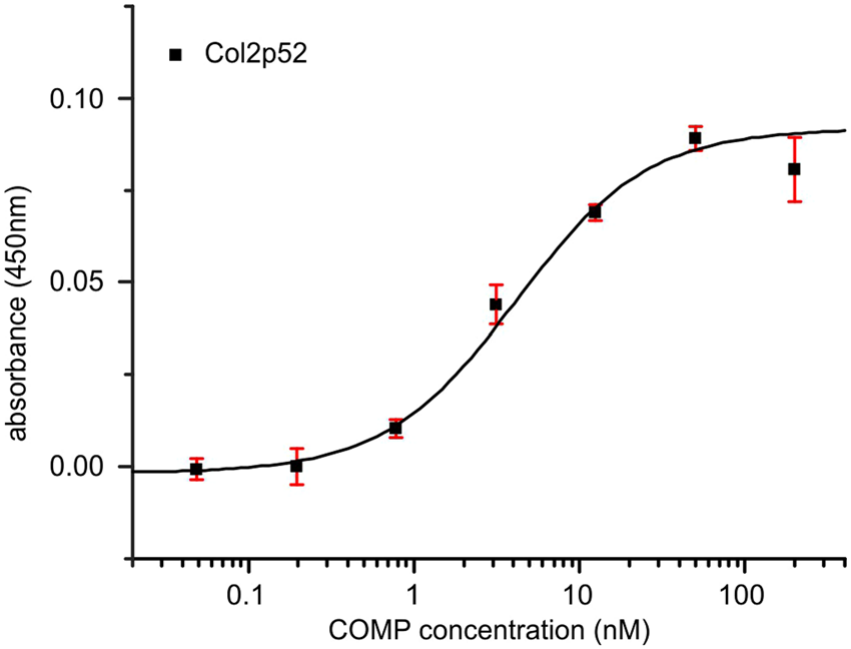
Binding of COMP to the C-terminal motif of collagen II contained in peptide 52. Peptide 52 of the collagen II foldon library was coated to an ELISA plate and incubated with COMP protein. Binding was detected with a specific anti-COMP antibody. Bars indicate standard deviation of triplicates. The peptide sequence is depicted in Table 1. The fitted K_D_ is 4.1 ± 0.8 nM.

From earlier studies it was known that COMP binds to the collagen helix at least twice^10^. We used our consensus sequence GXKGHR to search for additional binding sites on collagen II. Close to the C-terminal propeptide, the sequence _1128_GLKGHR_1133_ matches this consensus. We cloned, expressed, and purified the library peptide 52, which includes the GXKGHR motif in its unique sequence (GEAGEPGER**GLKGHR**GFTGLQGLPGPP). In ELISA-style binding assays this peptide bound with a good, although slightly reduced affinity (K_D_ 4.14 ± 0.77 nM) in comparison to peptide 5 (Fig. 5).

### Collagen XI also binds COMP

By searching other collagens for this collagen:COMP interaction sequence, we found the GXKGHR motif in collagen I, II, III, and additionally in collagen V, and XI (Table 3). The latter are to our knowledge currently not known to bind COMP. Interestingly, all motifs are located at the same positions in the collagenous domain. The N-terminal motifs always lies 28 GXX triplets from the start while the C-terminal motifs is placed around 27-28 triplets from the end of the collagen. The large numerical difference in numbering of collagen α1(V) is due to the big N-terminal domain of this particular chain.

**Table 3:** Binding sites in other collagens.

Collagen α1 (I)	_263_GMKGHR_268_ _1106_GIKGHR_1111_
Collagen α1 (II)	_285_GVKGHR_290_ _1128_GLKGHR_1133_
Collagen α1 (III)	_261_GMKGHR_266_ _1104_GIKGHR_1109_
Collagen α1 (V)	_640_GEKGHR_645_
Collagen α2 (V)	_297_GEKGHR_302_ _1140_GQKGHR_1145_
Collagen α1 (XI)	_610_GDKGHR_615_
Collagen α2 (XI)	_568_GEKGHR_573_

**Figure 6:**
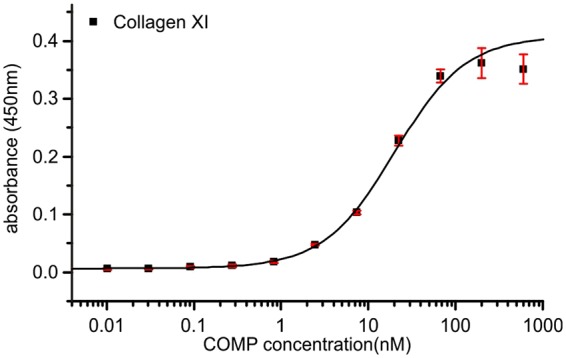
Binding of COMP to collagen type XI. Collagen XI was coated to an ELISA plate and incubated with COMP protein. Binding was detected with a specific anti-COMP antibody. Bars indicate standard deviation of triplicates. The peptide sequence is depicted in Table 1. The fitted K_D_ is 20.0 ± 3.0 nM.

We tested binding of COMP to collagen XI in an ELISA-style binding assay (Fig. 6). COMP binds to collagen XI, although the affinity is significantly lower compared to collagen I and II (20 nM ± 3 nM). The motifs found in collagen XI are GEKGHR and GDKGHR in the α1 chain and α2 chain, respectively. In contrast to the motifs in collagen I, II and III the X position is occupied by a negatively charged amino acids. It is tempting to speculate that this charge is responsible for the observed lower affinity.

### TSP-4 also interact with the GXKGHR motif

**Figure 7:**
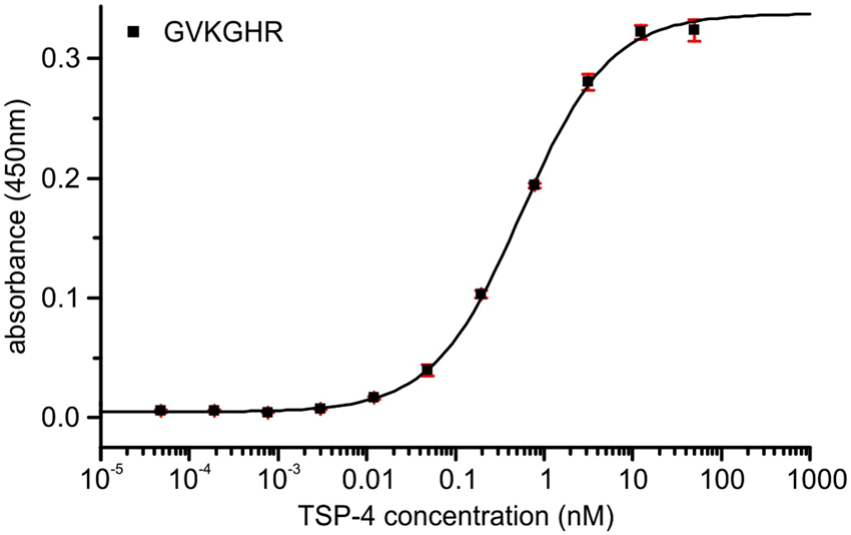
Binding of TSP4 to the GVKGHR motif. The minimal binding site GVKGHR expressed as a foldon construct was coated to an ELISA plate and incubated with TSP4. Binding was detected with a specific anti-TSP4 antibody. Bars indicate standard deviation of triplicates. The fitted K_D_ is 0.56 ± 0.05 nM.

COMP is a member of the Thrombospondin family. For TSP-4 it is known that it also binds collagens^22^. To test if our motif might be a common motif for collagen binding, we investigated whether TSP-4 binds to the GVKGHR peptide as well. Indeed, the affinity of TSP-4 (0.56 ± 0.05 nM) to our collagen model peptide was very similar to the affinity of COMP (Fig. 7).

### Docking of COMP to a collagen model peptide

**Figure 8:**
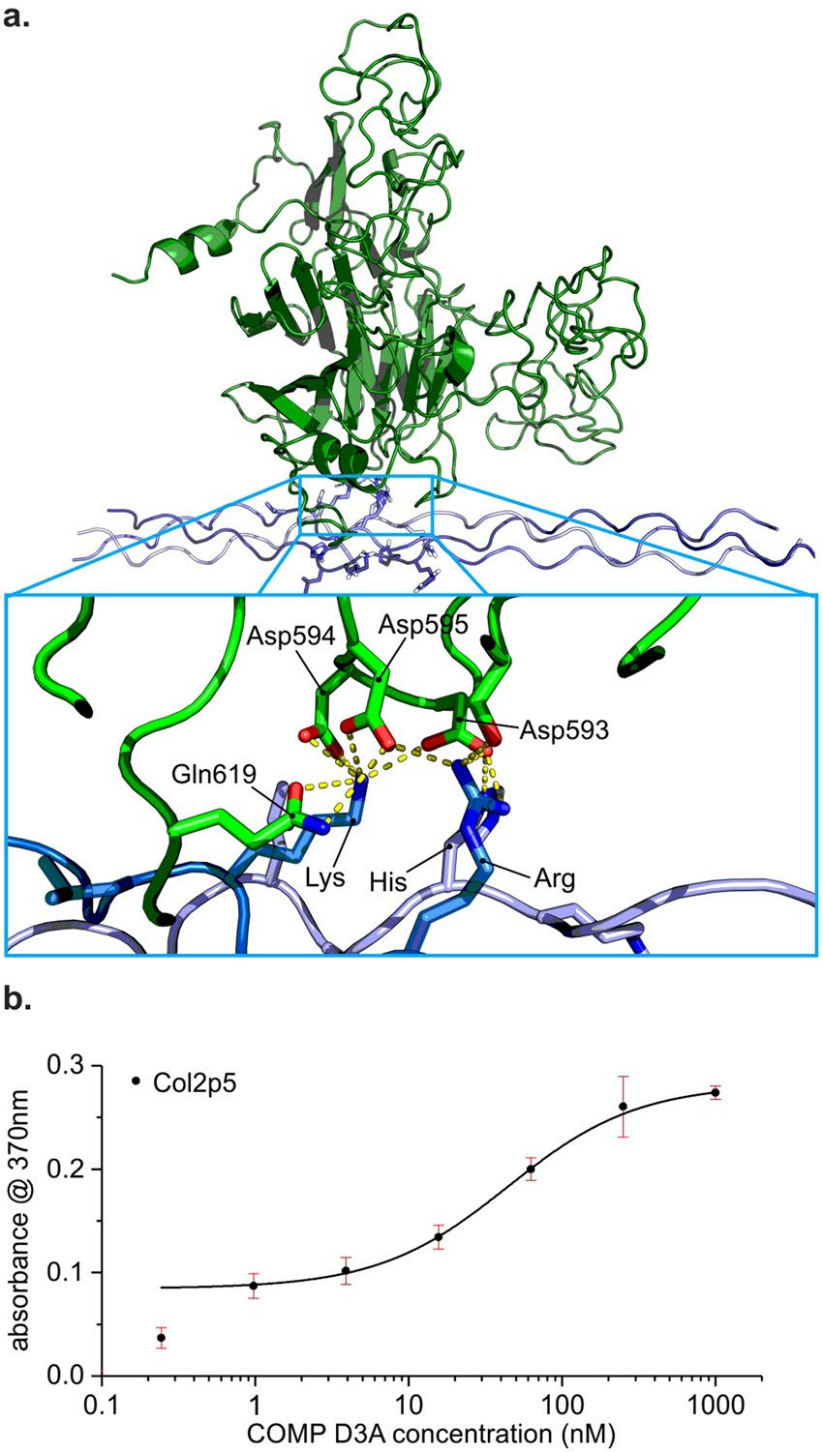
Docking of COMP to a collagen model peptide. (**a**) The COMP C-terminal domain (3FBY) was docked to our collagen model peptide carrying a GLKGHR motif using ClusPro2. The lysine and arginine residues of the leading and middle chain were defined as attractive. Best-rated models included the above shown complex, where the CMP interacts with the presumed MIDAS motif. Interestingly, this would displace the bound calcium ions and does not need a metal ion for binding. (**b**) ELISA-style-binding assay with Col2p5 and COMP D3A shows similar binding of COMP D3A to wt. Bars indicate standard deviation of triplicates.

To elucidate possible binding interfaces we used an unpublished crystal structure of a collagen peptide with the sequence Ac-(POG)_4_LKGHRGFTGLQG-(POG)_4_-NH_2_, which resembles the C-terminal COMP binding motif of collagen II. The statistics for data collection and structural solution of this peptide can be found in Table 4. Docking was performed with ClusPro and the best 10 models analysed by manual inspection. The models clustered into two groups, whereas one group with 7 of 10 structures, located the collagen helix around the aspartate triplet _593_DDD_595_, which was described earlier as a potential MIDAS motif^23^. Interestingly, the two calcium ions in the crystal structure would be replaced by the collagen side chains. Furthermore, no metal ion would be necessary for this interaction (Fig. 8). In the docked model, Asp_593_ of COMP form hydrogen bonds to the arginine and lysine of the middle strand (Fig. 8 dark blue) and the histidine of the middle chain (Fig. 8 light blue). Asp_594_ and Asp_595_ interact with the lysine of the middle strand. To test this model, we mutated all three aspartates to alanine residues and expressed and purified the protein. To our surprise the mutant behaved similar to the wildtype in binding assays (Fig. 8b), falsifying the proposed model.

**Table 4:** Data collection and refinement statistics.

Data collection	CMP
Beamline	X06DA (PXIII)/SLS
Wavelength (Å)	0.800
Space group	I2 (No. 5)
**Cell dimensions**	
*a*, *b*, *c* (Å)	31.97, 23.64, 107.94
*α, β*, *γ* (°)	90.00, 92.70, 90.00
Resolution (Å)	31.02–1.00 (1.06–1.00)
*Rsym*	0.046 (0.559)
*Rmeas*	0.024 (0.602)
*CC*_*1/2*_	1.00 (0.963)
*I*/σ*I*	18.92 (4.17)
Completeness (%)	99.7 (98.5)
Multiplicity	6.38 (6.25)
**Refinement**	
Resolution (Å)	31.02–11.00
No. reflections (test set)	43886 (598)
*R*_work/_*R*_free_ (%)	21.8/23.4
**No. atoms**	
Protein	686
Ligand/ion	17
Water	184
**B-factors**	
Protein	22.48
Ligand/ion	31.62
Water	34.89
**Ramachandran**	
Favoured (%)	100
Outliers (%)	0
**R.M.S deviations**	
Bond lengths (Å)	0.004
Bond angles (°)	1.25

Highest resolution shell is shown in parenthesis.

## Discussion

It is well known that cartilage oligomeric matrix protein (COMP) interacts with collagen type I and II^10^, the exact sequence however has been unknown up to now. We expressed overlapping peptides covering the first third of the collagen II helix, which is known to harbour at least one COMP binding site. The peptides were stabilised by flanking GPP repeats and a C-terminal T4 foldon domain. The overlapping peptides were designed similarly to the well-established collagen II toolkit^6^. Only the peptide 5 interacted specifically with pentameric COMP. We checked the trimeric nature of the collagen helix for peptide 5 and its derivatives via CD spectroscopy and could confirm a proper collagen helix at room temperature (Table 2 and Fig. S1). There may be further binding sites as we have only examined a library spanning the N-terminal part of collagen II and only checked the interacting peptides for proper triple-helix formation.

We investigated the unique sequence of peptide 5 (GLPGVKGHR) for binding by mutating single amino acids to proline (Fig. 4). The exchange of either the lysine or the arginine residue lead to a complete loss of binding at the measured concentrations, while the exchange of the histidine residue significantly reduced the affinity by several orders of magnitude. The exchange of the leucine and the valine residue did not alter affinity significantly and the shortened version GVKGHR bound collagen with similar affinity. Therefore, we propose that GXKGHR is a sufficient motif for collagen binding by COMP and crucial for high affinity interactions. The slight decrease in affinities observed with the shortened peptides in comparison to the peptide 5 might indicate some additional beneficial interactions further away from the core sequence.

This proposed core sequence occurs twice in collagen II. Firstly, as the originally found GVKGHR motif starting at position 285, and secondly as a GLKGHR motif starting at position 1128. This sequence is contained in the collagen II peptide 52, for which we also showed binding to COMP (Fig. 5).

To study how well these two sites are conserved during evolution, we reviewed all currently annotated orthologues of collagen II in the Ensemble database. Due to the repetitive sequence of the collagen helix, the overall conservation rates are high, with more than 80 percent identity amongst distant species (e.g. human and zebrafish). However, although the N- and C-terminal GXKGHR binding motif is completely conserved in all species, there are variation at the undefined position X_1_ (X in the motif above). N-terminally the GVKGHR motif in mammals (*Mammalia*) is converted to a GIKGHR motif in fish (*Sarcoptergyi*), while the GLKGHR at the C-terminus, is exchanged to GVKGHR in guinea pig and kangaroo rat, and to a GQKGHR motif in fish. This strongly supports the finding that the X_1_ position is not of strict importance for COMP binding, and that at least glutamine and isoleucine are tolerated at this position.

We then searched for other collagen types bearing a GXKGHR motif. We found this motif to be present in all classical fibrillar collagens (collagen I, II, III, V, and XI; compare Tab. 3), which might indicate that all fibrillar collagens are clients for COMP mediated fibril formation assistance. However we were not able to find our binding motif in the FACIT collagens, IX, XII, and XIV, which are also known to interact with COMP^12,24^. For collagen IX it is known that COMP’s C-terminal domain only interacts with the non-collagenous domains (NC 1–4)^11,24^. It is not unlikely that this interactions involves a different interface on COMP’s C-terminal domain. For collagen XII and XIV the exact binding sites are not yet determined, and initial studies indicated an involvement of the collagenous domain or its interspersed short NC domain. Whether COMP binds to a different collagen sequence or parts of the NC domains in these two collagens needs to be further investigated.

Interestingly, in collagen I, the GXKGHR motifs are present only in the α1 chain and not in the α2 chain. As collagen I is a heterotrimer build from two α1 chains and one α2 chain, there will be a significant difference in the COMP binding site of collagen I compared to the homotrimeric collagen II, i.e. collagen I only present two binding motifs instead of three. Although it is tempting to speculate that this implies a lower affinity of COMP to collagen I, two important aspects of collagen recognition have to be considered. Firstly, the exact stoichiometry of the collagen-COMP interaction is currently unknown. Other known collagen interfaces suggest a typical stoichiometry of 1 or 2 globular proteins per collagen triple helix. This is easy understandable, due to the tight packing of the collagen helix and the resulting steric hindrance for more than 2 binding partners (compare crystal structures of HSP47-collagen complex (PDB: 4AU2)^25^ or integrin α2-collagen complex (PDB: 4BJ3)^26^). Considering the relatively big and bulky size of the C-terminal domain and the adjacent T3 repeats (PDB: 3FBY^23^) it is more likely that only one COMP binds per collagen triple helix. This would also better explain the observed 1:5 ratio for COMP’s activity in fibril formation^10^. Secondly, collagen motifs are presented as three-dimensional epitopes. As the collagen superhelix turns 9° per amino acid, a typical interaction surface of collagen is generated by more than one chain. The prototypical collagen-binder integrin α2 (recognition sequence: GFOGER) for example recognises a glutamic acid, a phenylalanine, and an arginine on the trailing strand, and a phenylalanine, hydroxyproline, and arginine on the middle strand^26,27^. Similarly, the von Willebrand Factor recognises its collagen motif (GxRGQOGVMGFx) via the valine and phenylalanine from the leading strand, and the arginines from middle and trailing strand^28^. As neither the three-dimensional binding interface of collagen to COMP nor the stagger of the collagen I helix is known, we can currently not evaluate whether the absence of the GXKGHR motif from the α2 has any effect on the collagen binding. However, by conversion we could also conclude that our identified binding interface formed by a homotrimeric GXKGHR motif might also be formed by a heterotrimer with a different linear sequence, i.e. the lysine and arginine might come from different chains. Therefore, our current search motif potentially does not identify all possible interaction sites in heterotrimeric collagens – a problem present with all currently described binding motifs in collagens.

Although all thrombospondins share a common domain structure, only TSP-1, TSP-4 and COMP (TSP-5) are known to directly interact with collagenous domains^10,22,29^. The latter two proteins are also reported to form heterooligomers^30^. In the thrombospondin family, TSP3, TSP-4, and COMP are closely related (52%, for TSP-3 vs TSP-4, 46% TSP-4 vs COMP, 42% COMP vs TSP-3 sequence identity) and form a subgroup in the protein family, characterised by their ability to form (homo-)pentamers. It is tempting to speculate that this group shares a common binding motif to collagen, especially as the identity in the C-terminal region is even higher (83%, 86%, 80%). Indeed, we could show that TSP-4 binds to the same motif as COMP, indicating that this motif might be a general motif for collagen recognition by thrombospondins. Whether TSP-3 also binds collagen is to our knowledge not yet tested. TSP-1 is very different in its C-terminal domain (only approx. 50 percent sequence identity to TSP-3, -4 or COMP) and is more similar to TSP-2. Structurally, however, the C-terminal domain of TSP-1 (PDB: 1UX6^31^) and COMP (PDB: 3FBY^23^) are very similar and only differ by an rmsd of 0.89 Å, which might indicate that it binds collagen via a similar interface, despite their low sequence identity. Indeed very recently, others showed that TSP-1 interacts with the same binding site on collagens as COMP^32^.

Very interestingly, the GXKGHR motif is located in a region on collagen I and II, which seems to be a hotspot for collagen interactions. Recently, it was described that PEDF and heparin are binding in this region^33^. Furthermore, the lysine residue is known to be important for cross-linking collagen fibrils^34,35^. It is known that PEDF, as most collagen binding molecules, interfere with fibril formation *in vitro*^36^. The interaction of COMP with collagen might therefore not only regulate fibril formation, but also protect this region from prematurely binding to other molecules. Similarly, the interaction with COMP might prevent incorrect crosslinking of not finally assembled collagen fibrils.

Recently it was shown that COMP also interacts with collagens intracellularly^14^. Binding of COMP to collagen is important for collagen secretion and lack of COMP leads to intracellular retention of procollagen molecule, which is similar to the lack of HSP47^37^. Consequently, both proteins are described to be involved in fibrotic diseases. Recent studies showed that COMP ablation reduces negative effects of fibrotic condition in skin and liver^14,38^ and propose COMP as a good target for future anti-fibrotic drugs. Our current study reveals for the first time that COMP recognises fibrillar collagens specifically by a sequence motif not existent in other collagens. By inhibiting this interaction, a potential drug might specifically target the secretion of fibrillar collagens only, which are abundant in fibrotic tissues. This might be beneficial over the inhibition of HSP47, which also interacts with non-fibrillar collagens, at least collagen IV^15^.

Interestingly, the exact binding interface on the COMP site is still unknown. The susceptibility of the interaction to EDTA lead to the idea that divalent cations – presumably Zn ions – are important for the collagen interaction. However, only calcium ions were detected in the crystal structure of COMP^23^, or the structurally related TSP-1^31^ or TSP-2^39^, although a potential MIDAS motif was postulated in the former. Studies with a disease-relevant mutation in the C-terminal domain, showed an effect of mutations at position 587 on fibrillogenesis, which might be a result of defective collagen binding^17^. However, this mutation is more than 16 Å from the proposed MIDAS motif and a defective binding was no yet shown.

To get an idea of possible interaction sites, we docked a crystallised model peptide containing the C-terminal COMP binding motif of collagen II to the previously described crystal structure of COMP’s C-terminal domain. Interestingly, in most predicted complexes the collagen is recognised by three consecutive aspartate residues (Asp593–Asp595, Fig. 8) which were previously predicted to form a MIDAS motif^23^. The docking process does not support the addition of divalent cations and indeed none is necessary for the predicted interaction. Zn^2+^ is normally recognised by histidine, cysteine, or acidic residues (like aspartate or glutamate)^40^. Although there is a histidine present in the recognition motif on the collagen side, the more important lysine and arginine residues are unknown to play a role in metal coordination. By testing a generated triple mutant replacing all three aspartate residues by alanines, we could show that this potential MIDAS motif is not involved in the binding of COMP to collagen. This agrees with our earlier observation that zinc is not necessary for the interaction of COMP with collagen. Earlier studies identified the DDD motif to be important for the integration of thrombospondins into the extracellular matrix via direct TSP-TSP interactions^41,42^.

A final answer to this problem will best be provided by an experimental structural model of the complex, which is also of great importance for proper drug development. Our study characterised the collagen site and identified the minimal binding sequence necessary for COMP interaction. This should enable structural characterisation of the complex by high-resolution methods, which were previously hampered by the absence of defined collagen mimetic peptides.

In summary, our study showed for the first time, the minimal sequence requirements in collagen for COMP and TSP-4 binding. Interestingly, this motif (GXKGHR) overlaps with the recognition sequence of various other collagen binders (heparin, PEDF). Due to its close proximity to other important interactions sites, this sheds new light on the role of thrombospondin interaction for collagen fibril formation and matrix organization and will facilitate the further investigation of COMP as a fibrotic drug target.

## Experimental Procedures

### Generation of a foldon stabilized collagen II library

Peptide sequences were backtranslated into a DNA sequence and the sequence optimised for *E. coli* expression using the OPTIMZER webpage^43^. For facilitating cloning, an adapted golden gate assembly system was used^19,20^. Recognition sites for BsaI were attached to the 5′ and 3′ end of the sequence in that way that the recognition site of BsaI lies outside of the coding sequence (5′prime: TACATGGTCTCA|CGG, 3′prime: AGATGAGGTCTCA|GTCC; recognition site underlines, cut postion indicated by a |). The sequences were ordered as a forward and reverse oligo with a central overlapping region of approximately 20 bases, the oligos were hybridised and filled up with a Pfu polymerase. A pET based vector containing an N-terminal double Strep-tag followed by 12 GPP repeats and a C-terminal T4 foldon domain^21^ was altered to include an insert with two internal BsaI restriction sites (pCMP-3b, Fig. S2).

100 ng of vector and equimolar amounts of the double stranded DNA insert was incubated with 30 units BsaI-HF (NEB) and 1000 units of T4 Ligase (NEB) in a total volume of 15 µl and incubated in a thermocycler with 15 alternating cycles of 37 °C for 3 min and 16 °C for 4 min. After a final digestion at 50 °C for 5 min all enzymes were inactivated at 80 °C for 5 min. 5 µl of the reaction was transformed into DH5α and the plasmid DNA from single clones sequenced (GATC Biotech).

### Expression and purification of recombinant proteins

Collagen model peptides were expressed in BL21 (DE3) cells as described earlier^5^. Shortly, cultures inoculated from an overnight culture were initially allowed to grow at 37 °C and subsequently cooled down to 20 °C. At an OD_600_ of approx. 1.2, cells were induced by 1 mM IPTG and protein was expressed overnight at 20 °C. Cells were harvested, washed, and stored frozen at −20 °C. For protein purification, cells were ruptured in presence of 10 µg/ml DNAse in a cell disruptor (Constant Systems, UK), the lysis solution cleared by ultracentrifugation, and the supernatant loaded on a Strep-Tactin column (IBA). After washing with at least 10 column volumes, the bound protein was eluted with 2 mM desthiobiotin in Tris buffered saline (TBS). The proteins were dialysed against TBS overnight and stored in aliquots at −80 °C.

Recombinant COMP and TSP-4 was expressed as described before^12,44^. Essentially, COMP wt, D3A (Asp593Ala, Asp594Ala, Asp595Ala) and TSP-4 coding pCEP vectors were transfected into HEK293 (EBNA) cells and expressed under serum free conditions for 3 days. The supernatant was cleared by centrifugation and the protein purified via a Strep-Tactin column at room temperature. The protein was dialysed overnight against pure TBS and stored in aliquots at −80 °C.

### CD Spectroscopy and Dynamic Light Scattering (DLS)

CD spectra of the col2p5 peptide and its derivatives were measured using a Jasco J–715 equipped with a peltier-element cuvette cooler. The samples were heated at 1 °C/min in a 1 mm path length quartz cell at approx. 0.1 mg/ml. Thermal unfolding was measured in a range between 20 °C and 70–90 °C and specific ellipticity recorded at 210 nm ever 1 °C. Additionally, every 5 °C far-UV spectra (200–250 nm) were recorded in triplicates. Data was fitted with a 4 parameter logistic fit using Origin 2017^45^.

Dynamic light scattering (DLS) was measured with a Wyatt DynaPro Nanostar using the disposable cuvettes (UVettes, Eppendorf). Data was acquired as a mean of 5 acquisitions, measured each for 5 s at a fixed temperature of 20 °C. The laser was set to auto-attenuation and the data analysed with the Dynamics software from Wyatt. Graph show the cumulant radius versus time for COMP with indicated ZnCl_2_ concentrations.

### ELISA-Style Binding Assays

For ELISA-style binding assays, rat tail collagen I (BD Bioscience) or the indicated collagen model peptides were diluted in TBS and coated at 10 µg/ml (500 ng per well) overnight at 4 °C. Further steps were performed at room temperature. After washing with TBS-T (TBS containing 0.05% (vol/vol) Tween-20), plates were blocked for 1 h with 1% (wt/vol) BSA in TBS. Strep-tagged COMP was added at indicated concentrations in TBS-T containing 0.25% (wt/vol) BSA) and incubated for 1 h. For assays with addition of cations pure TBS-T was used as interaction buffer. After washing away unbound protein with TBS-T, bound COMP was detected with a 1:3000 dilution of a specific polyclonal antibody against bovine COMP in interaction buffer. The antibody was raised in rabbit against bovine COMP purified from articular cartilage and characterised before in various studies^46–48^. The TSP-4 antibody was raised in guinea pigs and was previously described and characterised^30,49^. The primary antibodies were detected with an HRP–conjugated swine anti-rabbit (DAKO, P0399) or rabbit anti-guinea pig antibody (Sigma, A5545). Bound secondary antibodies were visualised with tetramethylbenzidine as substrate, the reaction was either measured directly at 370 nm or stopped with 10% H_2_SO_4_ and measured at 450 nm. Data were analysed using Origin 2017 and fitted using a four-parameter logistic model^45^.

### Crystallization and Structure Solution of the collagen model peptide and Docking

A collagen model peptide coding for the N-terminal binding site of COMP, flanked by 4 GPOs (Ac-POG POG POG POG LKG HRG FTG LQG POG POG POG POG-NH_2_) were ordered from Peptide 2.0 (USA). The peptide was dissolved at approximately 250 µM in Water and mixed 1:2, 1:1 and 2:1 in various crystallisation screens using a Mosquito Nanodrop. Crystals were finally grown in 1.5 M Ammonium sulphate and 0.1 M BIS-TRIS propane pH 7.0 at 20 °C by vapour diffusion, cryoprotected with the addition of 20% glycerol to the mother liquor and flash frozen in liquid nitrogen. Data up to 1.4 Å were collected at beamline X06DA, Swiss Light Source (Paul Scherer Institute, Villigen, Switzerland). The data were processed in I2 using the program iMosflm^50^ and the Pointles-Aimless-cTruncate^51,52^ pipeline from within CCP4i^53^ and the structure solved by molecular replacement using PHASER^54^, using a collagen model peptide solved earlier (PDB: 4AXY^54^) as search model. The initial model was completely rebuilt manually using COOT^55^ and refined using iterative cycles of model building and refinement with phenix.refine^56^. The data collection and model refinement statistics can be found in Table 4. The model and diffraction data, was deposited at the PDBe under the accession number 6HG7. Docking was performed using the ClusPro Server 2.0^57,58^ using parts of the model of the COMP’s C-terminal domain (PDB Code 3FBY^23^, residues Asp_530_–Ala_757_). The lysine, arginine, and histidine residue of the trailing chain and the lysine residue of the leading chain of the collagen model peptide were defined to be attractive in the algorithm. These four amino acids are accessible from one side of the helix and might, thereby constitute one binding interface. In the generated models, the isolated C-terminal domain was replaced by the full-length model (3FBY) to detect possible clashes of the collagen molecule with the TSP repeats.

## Electronic supplementary material

Supplementary information
