# Supplementary material for: COMP and TSP-4 interact specifically with the novel GXKGHR motif only found in fibrillar collagens
Source: Sci Rep. 2018 Nov 21;8:17187. doi: 10.1038/s41598-018-35447-8 (PMC6249252; doi:10.1038/s41598-018-35447-8)
Supplement: Supplementary file 1 — Supplementary information [file 41598_2018_35447_MOESM1_ESM.pdf]

SUPPLEMENTAL INFORMATION

FOR

COMP and TSP-4 interact specifically with the novel GXKGHR motif only found in fibrillar collagens.

Authors and Affiliations

Jan M. Gebauer[1*], Anna Köhler[2], Helen Dietmar[2], Monika Gompert[1], Ines Neundorf[1], Frank Zaucke[2,3], Manuel Koch[2,4] and Ulrich Baumann[1]

[1]Institute of Biochemistry, University of Cologne, Cologne, Germany.
[2]Center for Biochemistry, Faculty of Medicine and University Hospital Cologne, University of Cologne, Cologne, Germany.
[3]Dr. Rolf Schwiete Research Unit for Osteoarthritis, Orthopedic University Hospital Friedrichsheim gGmbH, Frankfurt, Germany.
[4]Institute for Dental Research and Oral Musculoskeletal Biology, Faculty of Medicine and University Hospital Cologne, University of Cologne, Cologne, Germany.

[*]To whom correspondence should be addressed: jan.gebauer@uni-koeln.de, Institute of Biochemistry, University of Cologne, Zülpicher Str. 47, 50674 Cologne, Germany

Figure S1

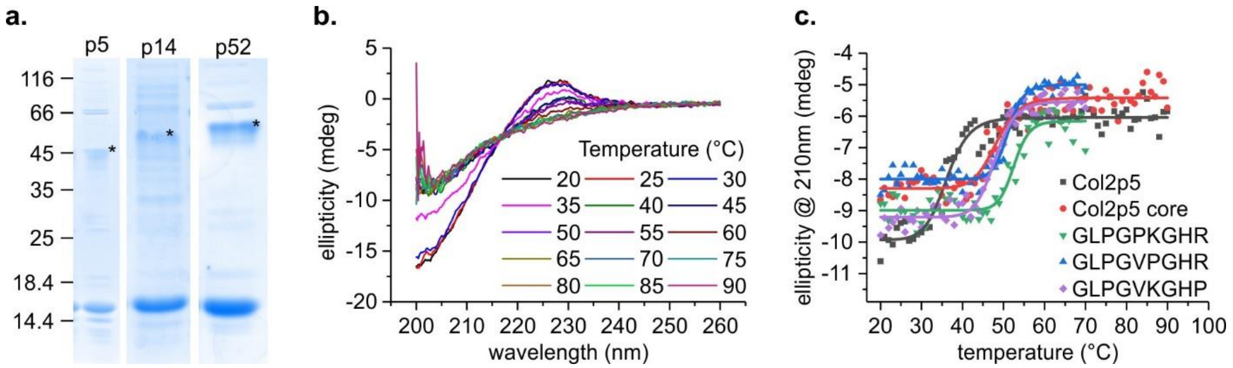

Fig. S1. Collagen model peptides run partially as SDS stable trimers and form proper triple helices. (a) Purified collagen model peptides run as partially SDS-stable trimers (asterix). The calculated molecular mass of the monomers is approximately 13 kDa. (b-c) CD spectrometry of the collagen II peptide 5 and its derivatives showed a stable collagen helix. The estimated melting temperature of the peptides can be obtained by measuring the increase of ellipticity at 210nm (Boudko et al. 2002) (b) Full CD spectra for the collagen II peptide 5, showing a stable signal up to 30 °C (c) melting curves measured via CD spectroscopy at 210 nm for the col2p5 derivatives. The melting point for the full-length col2p5 is at 36±0.5°C (Col2p5) while all the other are at approximately 50°C (See table 2 for details) .

Figure S2

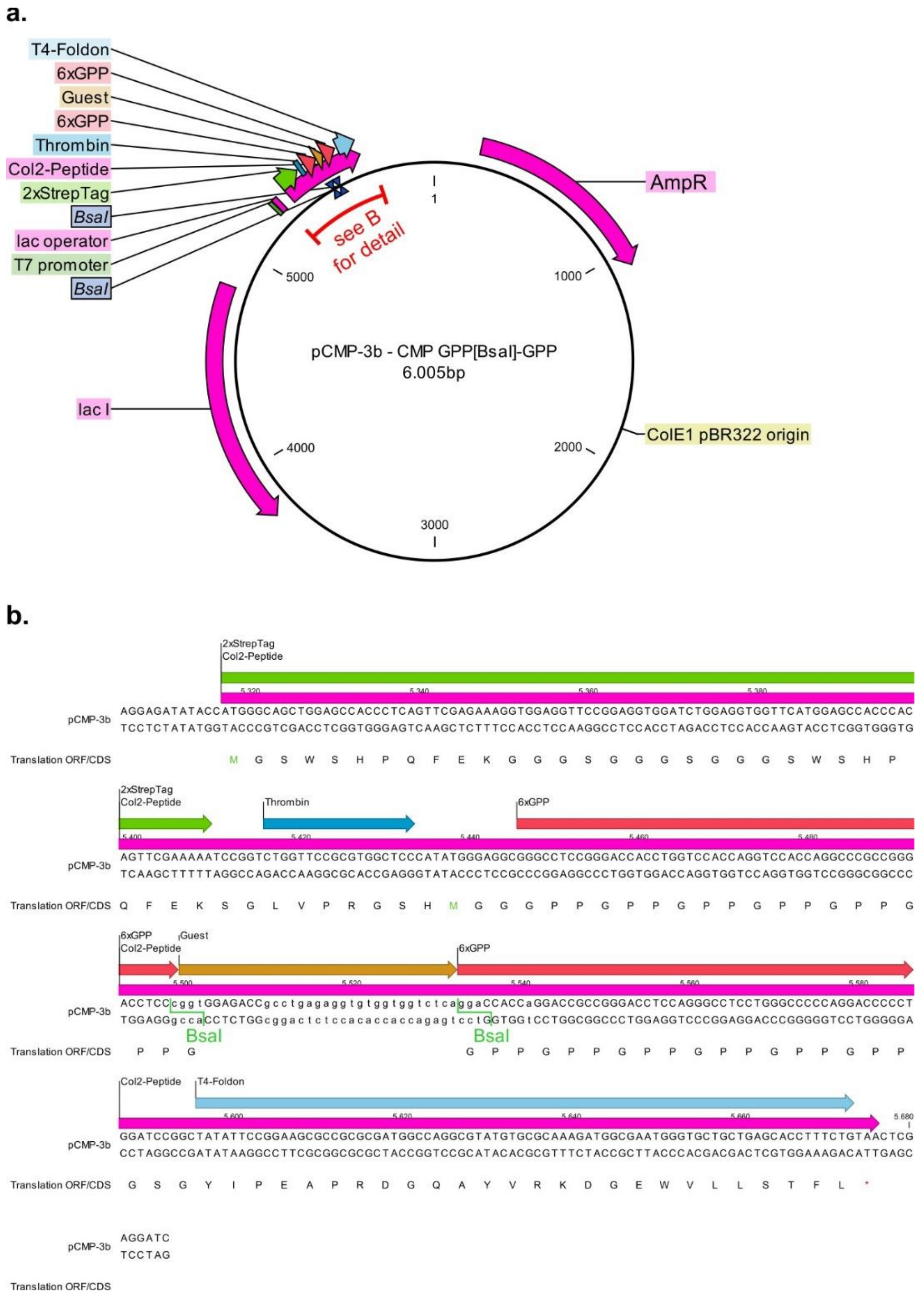

Fig. S2. pCMP-3b – a vector for efficient cloning of collagne peptides. (a) Overview of the pET-based vector. (b) Detailed view of the Golden-Gate insertion site of pCMP-3b (indicated in (a) by the red arc).

References

Boudko, Sergei, Sabine Frank, Richard A Kammerer, Jörg Stetefeld, Therese Schulthess, Ruth Landwehr, Ariel Lustig, Hans Peter Bächinger, and Jürgen Engel. 2002. “Nucleation and Propagation of the Collagen Triple Helix in Single-Chain and Trimerized Peptides: Transition from Third to First Order Kinetics.” *Journal of Molecular Biology* 317 (3): 459–70. https://doi.org/10.1006/jmbi.2002.5439.
